# Effect of Drilling Parameters on Machining Performance in Drilling Polytetrafluoroethylene

**DOI:** 10.3390/ma15196922

**Published:** 2022-10-06

**Authors:** Jing Ni, Xiaotian Zeng, M. S. H. Al-Furjan, Huijun Zhao, Liming Guan, Zhi Cui, Lidong Han

**Affiliations:** 1 School of Mechanical Engineering, Hangzhou Dianzi University, Hangzhou 310018, China; 2 State Key Laboratory of Mechanics and Control of Mechanical Structures, Nanjing University of Aeronautics and Astronautics, Nanjing 210016, China; 3 School of Mechanical Engineering, Hangzhou Dianzi University Information Engineering College, Hangzhou 311305, China

**Keywords:** polytetrafluoroethylene, cutting parameters, thrust, torque, drilling temperature, surface roughness

## Abstract

Polytetrafluoroethylene (PTFE) plays an important role in semiconductor manufacturing. It is an important processing material for the key sealing components in the field of immersion lithography. The lack of research related to the mechanical processing of PTFE leads to many challenges in producing complex parts. This paper conducted a drilling experiment on PTFE. The effect of cutting parameters on the drilling performance was investigated. Thrust, torque, surface roughness, and drilling temperature were used to evaluate the influence of cutting parameters on drilling performance. In addition, the empirical mathematical models of thrust and torque were developed using analysis of variance (ANOVA). The results indicated that the spindle speed had the most important effect on the thrust and the feed rate had the most significant effect on the torque. The lowest values of thrust and torque were, respectively, 22.64 N and 0.12 Nm, achieved in the case of spindle speed of 5000 rev/min, and feed rate of 50 mm/min. The surface quality is also best at this cutting parameter. Studies have shown that higher spindle speeds with lower feed rates are ideal parameters for improving the drilling performance and machining quality of PTFE. In addition, it was found that the temperature differences due to different drilling depths were related to chip accumulation. Surface roughness inconsistencies at various locations in the inner wall of the hole were influenced by chip adhesion during machining. This paper provides a suggestion for optimizing cutting parameters and hole quality.

## 1. Introduction

Polytetrafluoroethylene (PTFE) is a synthetic fluoropolymer, classified among thermoplastics [1,2]. It has many excellent characteristics such as high-temperature stability, chemical resistance, and low coefficient of friction [3,4]. Hence, it is widely used in semiconductor manufacturing equipment, especially as critical preparation material for the key sealing parts of immersion lithography. PTFE is typically manufactured with processes such as sintering or high-velocity compaction [5]. However, PTFE parts produced by molding techniques often do not meet the required dimensional tolerances and assembly requirements. In order to improve accuracy, machining such as drilling is used to further process these materials [6,7]. Due to the low intermolecular forces, PTFE suffers from poor creep resistance as well as low stiffness and yield stress [8]. The machining performance of PTFE is significantly affected by cutting parameters. With improper cutting parameters, it is difficult to accurately process PTFE, resulting in burr, edge overcutting, inner surface damage, and other problems [9].

In recent years, the relationship between machining performance and cutting parameters of polymers and polymer composites has been extensively studied, among which thrust [9], torque [10], and surface roughness [11] are often paid more attention as indicators to evaluate machining properties. Rubio et al. compared the influence of cutting parameters on the machining performance of engineering plastics such as ultra-high molecular weight polyethylene (UHMWPE), polyformaldehyde (POM), and polytetrafluoroethylene (PTFE) [12]. They found that the surface roughness of PTFE is minimum at a lower spindle speed and feed rate and the minimum thrust values of the three materials are obtained at a high level of spindle speed and a low level of feed speed. Cui et al. studied the effect of cutting parameters on cutting force and surface quality during orthogonal cutting pure PTFE [13]. The results showed that cutting speed had the most obvious effect on cutting force while the cutting speed had the most significant effect on surface roughness. Gan et al. introduced cooling technology during milling of PTFE [14]. They found that cryogenic cooling could make PTFE show little viscous behavior. This leads to the increase of the cutting force. Ramesh et al. focused on the effect of drilling parameters on thrust force in the drilling of sisal-glass fiber reinforced polymer composite laminates [15]. The quadratic response models are developed by using response surface methodology (RSM) to predict the influence of cutting parameters on thrust force. The results showed that the thrust force is significantly influenced by feed rate, followed by spindle speed. The influence of drilling parameters on the thrust force and torque during traditional drilling of glass fiber reinforced composites was studied by Mohan et al. The effect of spindle speed has more significance concerning thrust by Taguchi analysis and analysis of variance (ANOVA) experiment [16]. Susac Florin et al. investigated the effect of machining parameters on the hole quality in the drilling of polyethylene, polyamide, and polyacetale [17]. The results indicated that the surface roughness increases with increasing spindle speed. The ideal surface roughness can be obtained by drilling at a lower spindle speed and a higher feed rate. Yasar, Nafiz et al. pointed out that the surface roughness value of carbon fiber reinforced polymer composites increases with the increase of the feed rate in drilling [18]. The feed rate is determined to be the most significant factor affecting the surface roughness by the analysis of variance.

In addition, due to the low thermal conductivity of PTFE, the high temperatures generated during the drilling process can soften the material, resulting in machining defects. So, the temperature is also an important evaluation index of machining performance. At present, the relationship between cutting parameters and temperature of PTFE and other thermoplastic materials has been reported. Alper Uysal et al. investigated the effect of drilling angle, cutting speed, and feed on the cutting temperature of drills in drilling of pure polypropylene, using a two-wire infrared thermometer [19]. The results indicated that the cutting temperature decreased with increasing feed during drilling polymer material. Under the condition of constant cutting speed, more heat is transferred from the drill to the polymer material. Chang et al. explored the influence of cutting parameters on the temperature of polyetheretherketone (PEEK); an infrared thermography was used to record the temperature variation within the drilling area [20]. Experimental results showed that the temperature is inverse to the feed rate. Khashaba et al. explored the effect of spindle speed and feed rate on temperature in drilling GFRP composites [21]. Two temperature measurement methods are used, one is to use a thermal infrared camera, and the other is to use a K-type thermocouple, which is placed in a cooling hole inside the drill bit. The results showed that compared with the feed rate, the spindle speed has a more significant influence on the temperature than the feed.

So far, some studies on the cutting performance of polymers and polymer matrix composites have been reported, but the effects of cutting process parameters on machining properties such as thrust, torque, surface roughness, and cutting temperature have not been considered comprehensively. Although polytetrafluoroethylene (PTFE) is widely used, few studies have focused on the drilling of PTFE materials. Therefore, thrust, torque, workpiece temperature, and surface roughness are used as indicators to characterize the machining performance in this paper. Small hole dry drilling experiments were conducted on PTFE. The effect of cutting parameters such as spindle speed and feed rate on the machining performance of PTFE was systematically investigated.

## 2. Experimental Procedure

### 2.1. Workpiece and Cutting Tool

The PTFE workpiece was purchased from Shanghai Valqua Fluorocarbon Products Co., Ltd. Based on the relevant technical documents given by the supplier, the physical and mechanical properties of the workpiece are listed in Table 1.

**Figure 1:**
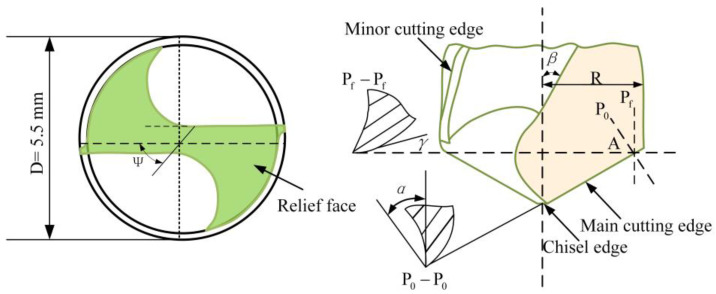
The geometrical details of cutting tool.

Rod PTFE workpieces with a diameter of 15 mm and a length of 30 mm were used for the experiment. In terms of cutting tools, uncoated double-edge high-speed steel twist drills with a diameter of 5.5 mm were selected. For a double-edge HSS twist drill, there are two main cutting edges, two minor cutting edges, and one chisel edge, which generate thrust and torque during the drilling. The twist drill of the chisel edge is in the shape of a cross. The geometrical details of the cutting tool are shown in Figure 1. The geometrical parameters of the twist drill are shown in Table 2.

### 2.2. Experimental Details

**Figure 2:**
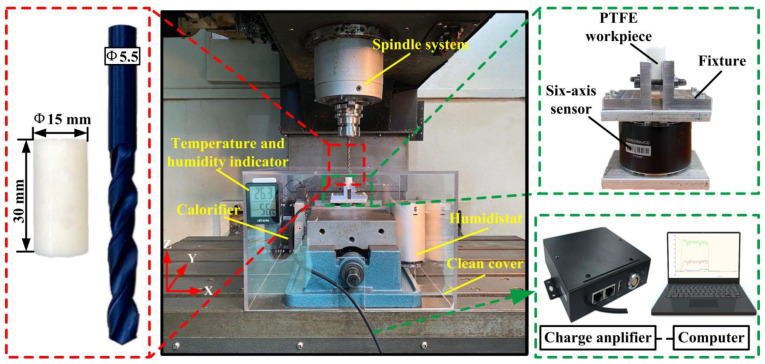
Experimental system setup.

The experiment was carried out on a FEELEVM-32SA three-axis CNC vertical machining center with a maximum spindle speed of 10,000 rev/min. In order to ensure that the processing area is in a constant temperature and humidity state, a self-made clean cover was used to isolate the processing area from the external environment. In addition, this measure also contributes to the collection of chips. The drilling test was carried out under dry machining conditions without a cooling system. The experimental system diagram is shown in Figure 2. The ME-K6D40 six-dimensional force sensor was used for signal acquisition of thrust and torque. The signals measured during drilling were transmitted to the computer through the signal amplifier HSGD6X-EC.

In order to investigate the effect of drilling parameters on the drilling performance of PTFE, drilling experiments at different spindle speeds and feed rates were carried out. A full factorial design with two factors and five levels was adopted in this experiment. The combination of five spindle speeds and five feed rates is listed in Table 3. Before each test, the tool and workpiece were reset to ensure that no other factors except the drilling parameters would interfere with the drilling.

**Figure 3:**
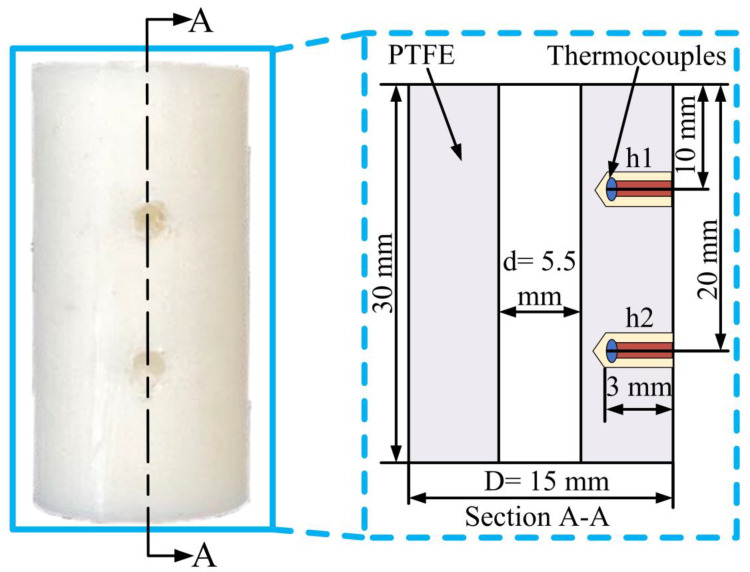
Thermocouple hole size and preset position.

In this experiment, two small holes of size Φ1 × 3 were machined on the workpiece, and the two small holes were named h1 and h2, respectively. Thermocouples with a measurement range of 0–122 °C are mounted on the side of the workpiece through these two holes to achieve accurate temperature acquisition during drilling. The specific location and related parameters of thermocouple holes are shown in Figure 3. The measurement of temperatures could be affected by the low thermal conductivity of PTFE and the distance between the tip of thermocouple and the inner wall of the hole. Therefore, the measurement of temperature does not stop immediately at the end of drilling. The measurement of temperature is stopped only when the temperature measured by the two thermocouple drops. This ensures sufficient time for the temperature generated by drilling to be transmitted to the thermocouple, thereby mitigating the effect of response delay on the temperature difference between the two thermocouples. The temperature data were stored and recorded by the YET-620L thermocouple temperature logger. The drilling temperature mentioned in this study refers to the maximum value of temperature measured by the thermocouple.

The workpiece was cut in half along the axis and the surface roughness of the inner wall is measured. The surface roughness (Ra) of the holes was measured by the surface roughness measurement instrument Mitutoyo SJ-210 with a sampling length (cut-off length) of 0.8 mm. The traveling speed of the prob was 0.5 mm/s when measuring Ra of the workpiece. Each sample was measured three times and the average value was taken as the surface roughness value.

## 3. Results and Discussion

### 3.1. Effect of Drilling Parameters on Thrust and Torque

#### 3.1.1. Analysis of Thrust and Torque Variation Process

**Figure 4:**
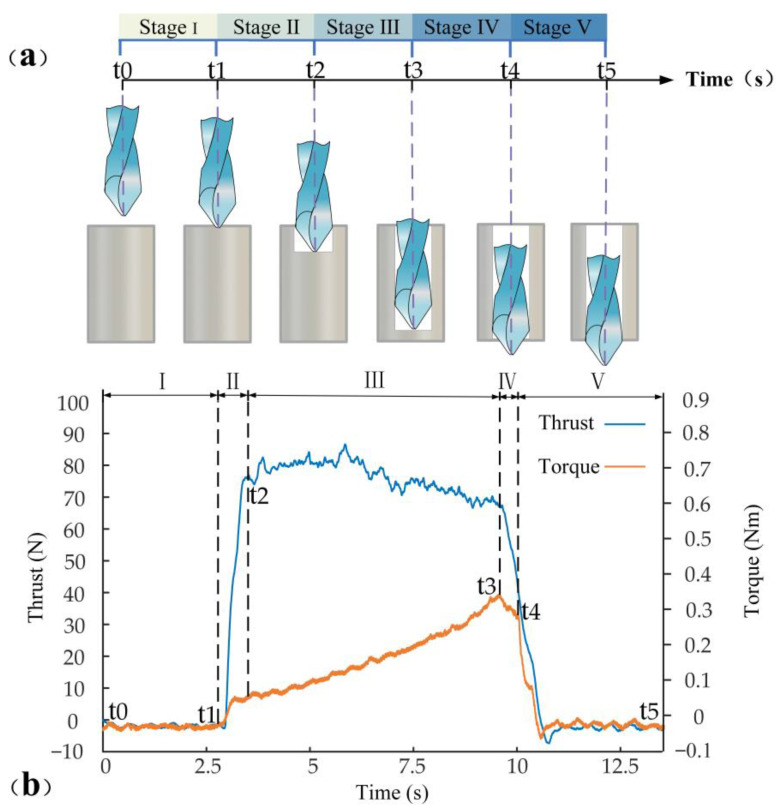
Drilling process diagram: (**a**) Schematic diagram of drilling processing stage; (**b**) Curve of thrust and torque variation.

The drilling process of PTFE was divided into five stages, and the variation of thrust and torque in each stage of PTFE drilling is shown in Figure 4.

Stage Ⅰ is the preparation process for the drilling process. The drill bit starts to rotate but has not yet touched the PTFE workpiece material. At this time, the cutting force and torque are both zero.

Stage Ⅱ is the process in which each cutting edge of the bit was involved successively in the machining. At t_1_, the chisel edge of the drill bit approaches the surface of PTFE and the thrust force starts to increase rapidly. Since the speed of the chisel edge is almost zero, it creates a squeezing effect on the workpiece surface, which increases thrust. As the drilling depth increases, the main cutting edge of the drill begins to participate in the drilling. The torque and thrust increase with cutting volume due to the increased drill diameter in contact with PTFE. At t2, the main cutting edge of the bit is completely drilled into the PTFE.

Stage Ⅲ is the stable drilling stage. At this stage, the cutting edges of the drill bit are fully engaged with the material and the thrust curve remains stable. The contact length between the drill bit and workpiece increases with the increase of feed speed, increasing torque.

Stage Ⅳ is the process in which the bit is about to be drilled. At t3, the chisel edge approaches the bottom of the workpiece, when the unmachined PTFE layer is already very thin. The reduction in thickness leads to weaker material strength, which further leads to less resistance to overcome the elastic deformation of the material. Therefore, thrust and torque are suddenly reduced in this stage.

Stage Ⅴ is the stage when the drill bit drills out the workpiece. At t4, the thrust and torque decrease rapidly as the chisel edge drills through the PTFE. At t5, the thrust and torque rapidly decrease to zero since all cutting edges are drilled out of PTFE.

#### 3.1.2. Effect of Spindle Speed on Thrust and Torque

**Figure 5:**
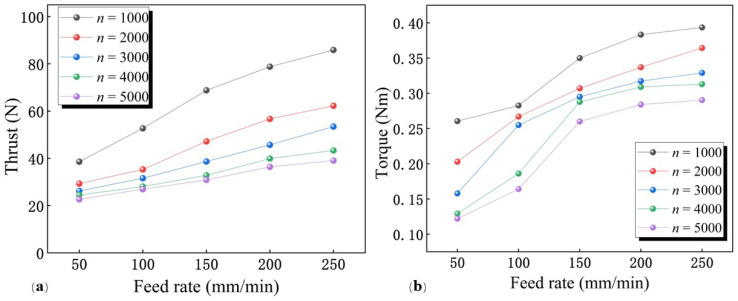
Variation curves of thrust and torque at different cutting parameters. (**a**) Thrust; (**b**) Torque.

The variation of thrust and torque with respect to the drilling parameters is shown in Figure 5. Both the thrust and torque decrease with increasing spindle speed under different feed rates. In the case of a feed rate of 250 mm/min, the thrust at 1000 rev/min increased by 47.31 N, compared with that at 5000 rev/min, reaching a maximum value of 85.90 N. 

This is because increasing the spindle speed while maintaining a constant feed rate will result in a corresponding reduction in cutting volume per revolution. The increase in spindle speed increases the friction heat per unit time, which leads to the increase of cutting heat [22]. It is difficult to carry away cutting heat through the chips due to the difficulty of chip evacuation during the drilling. The low thermal conductivity of PTFE also makes it difficult to transfer cutting heat through the workpiece. Since PTFE is a temperature-sensitive material for flow stress, an increase in temperature will lead to a decrease in its deformation resistance. Therefore, the thrust decreases as the spindle speed increases.

Ye et al. considered that with the increase in temperature, the transfer film of the PTFE machining surface was easier to form and became thicker, which would lead to a decrease in the average friction coefficient between the tool and the workpiece [23]. The increase in spindle speed will raise the temperature and result in a decrease in the friction coefficient. The reduction in the coefficient of friction between the tool and the workpiece results in reduced frictional resistance, which further leads to a reduction in thrust force.

In addition, the phenomenon that thrust decreases with the increasing spindle speed in Figure 5a may also be related to the “recovery of elastic“ effect of the polymer materials [24]. The “elastic recovery” effect of PTFE is more significant due to its low modulus of elasticity. This leads to easier contact between the machined surface and the flank face of the drill. The thrust will be increased by the friction and extrusion of the flank surface. Compared with low spindle speed, the machining surface is not fully recovered and is in contact with the flank face when drilling with higher spindle speed. Therefore, the extrusion and friction between the machined face and flank face will decrease, resulting in a lower thrust force as the spindle speed increases.

The trend of torque as a function of drilling parameters is shown in Figure 5b. When the feed rate is 250 mm/min, compared with 5000 rev/min, the torque increases by 0.11 Nm at a spindle speed of 1000 rev/min and reaches a maximum value of 0.39 Nm. During the drilling process, the friction between the hole wall and the drill bit as well as the chip will have an impact on the torque. Due to the “elastic recovery” effect, the friction between the drill bit and the hole wall is reduced at high spindle speeds, leading to a reduction in frictional torque. Therefore, the torque decreases as the spindle speed increases.

#### 3.1.3. Effect of Feed Rate on Thrust and Torque

It could be observed in Figure 5 that both thrust and torque decrease with decreasing feed rate. When the spindle speed was 5000 Rev/min, the thrust decreased by 42%, reaching the minimum 22.64 N, as the feed rate decreased from 250 to 50 mm/min.

**Figure 6:**
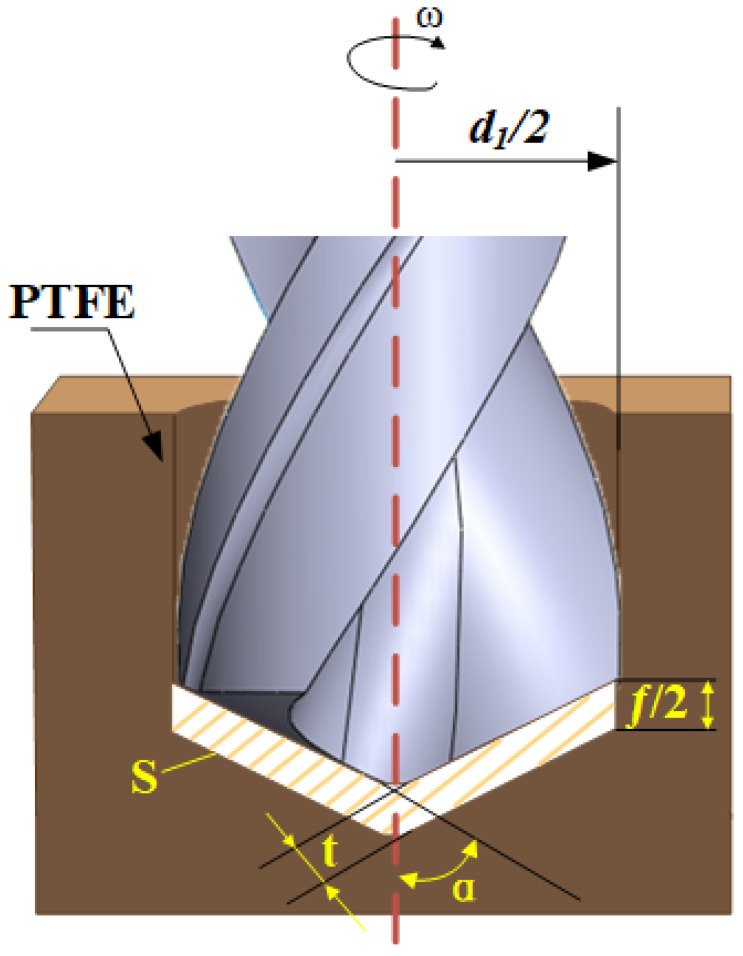
Diagram of drilling principle.

According to the drilling principle, the magnitude of thrust is related to the cutting thickness and area [25]. The drilling principle is shown in Figure 6.

The relationship between feed rate, spindle speed, and feed per revolution is shown in Equation (1):

(1) $$V_{f}=nf=2nf_{z}$$

where *V_f_* is the feed rate; *n* is the spindle speed; *f* is the feed per revolution. Since the HSS twist drill has two cutting edges, the feed per tooth *f*_z_ is *f*/2. According to Equation (1) and the drilling processing mechanism, *t* can be calculated as:

(2) $$t=f_{z}\sin\alpha =\frac{V_{f}}{2n}$$

where *t* is the cutting thickness. Finally, *S* can be deduced from Equations (1) and (2):

(3) $$S=t\cdot \frac{d_{1}}{\sin\alpha }=\frac{fd_{1}}{2}=\frac{V_{f}d_{1}}{2n}$$

where *S* is the cutting area per tooth when the cutting edge is fully engaged in the cutting action; *d*_1_ is the diameter of the drill bit. Combining Equations (1)–(3), the area and thickness of the material removed by the tool increase with the increase of the feed rate under the same spindle speed. In this case, the deformation resistance of the workpiece increases, resulting in a greater thrust required to remove the materials. Therefore, the thrust force increases with increasing feed rate.

In addition, the chip generated during the machining process will also affect the thrust. The morphology of the chips under different machining parameters is listed in Table 4. By observing the chip morphology, it is found that the chip shape changed from ribbon and node shape to pagoda shape gradually with the increase of feed rate. This makes chip removal difficult during processing, which increases the interaction force between the tool, workpiece, and chips, resulting in increased thrust.

Torque increases with increasing feed speed while keeping the spindle speed constant. This phenomenon can be attributed to the increase in material removal rate caused by the increase in feed rate. This leads to more chip generation and increased friction between the chip and the hole wall, resulting in increased frictional torque.

#### 3.1.4. Mathematical Model of Thrust and Torque

In order to establish a mathematical model of thrust and torque, the obtained experimental data were subjected to analysis of variance (ANOVA). The relative importance of the drilling parameters concerning thrust and torque was investigated by analysis of variance. The ANOVA results for thrust and torque at a confidence level of 95% are shown in Table 5 and Table 6. The values of the sum of squared deviations (SS), DF, mean square (MS), F-value, and *p*-value and percentage of contribution of each parameter are calculated. A *p*-value greater than 0.05 indicates that the drilling parameter has no significant influence on thrust and torque.

Table 5 shows the ANOVA results for thrust. The model F-value of 279.49 in Table 5 implies that the model is significant for thrust. The *p*-value of the model is less than 0.0001, which indicates that the model term is significant. Spindle speed is the most important factor affecting the thrust with a contribution of 46.8%; the next important effect is the feed rate with a contribution of 39.8%. 

Table 6 shows the ANOVA results for torque. From Table 6, it can be seen that the F-value of the model is 176.67, which indicates that the model is still significant. The feed speed has the largest effect on the torque with a contribution of 65%, followed by the spindle speed, which has a significant effect on the torque with a contribution of 26.7%.

In this study, the relationship between each factor (drilling parameters) and output (thrust, torque) was simulated using quadratic regression based on ANOVA. To improve the accuracy of the mathematical model, terms with *p*-values less than 0.05 were ignored. The regression equations of the thrust and torque models are shown in Equations (4) and (5), with coefficients of determination R-squared of 0.9824,0.9619 listed in Table 5 and Table 6.

(4) $$\mathrm{Thrust}=+44.46535-0.016798n+0.26420V_{f}-3.89275E-0.5nV_{f}+2.43998E-0.6n^{2}$$

(5) $$\mathrm{Torque}=+16.24400-2.70400E-0.3n+0.19745V_{f}-3.76686E-0.4V_{f}{}^{2}$$

Six validation experiments were carried out to verify the validation for the proposed model. The comparison result between the predicted values and the actual values are shown in Table 7 and Table 8. As listed in the table, for thrust and torque, the maximum relative errors between the experimental results and the prediction model are 10.7% and 13.7%, respectively.

### 3.2. Effect of Drilling Parameters on Drilling Temperature

**Figure 7:**
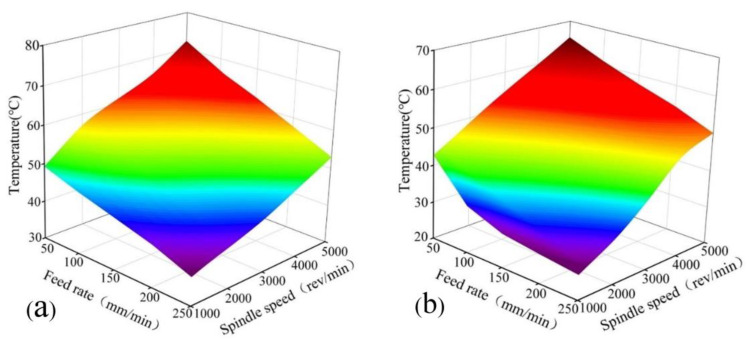
Three-dimensional graph of drilling temperature under different drilling parameters. (**a**) at h1; (**b**) at h2.

PTFE has the characteristics of low thermal conductivity and high thermal expansion coefficient. With the drilling of PTFE, it is easy to produce high temperatures, resulting in workpiece deformation, thus affecting the accuracy of the workpiece. The influence of drilling parameters on drilling temperature at different positions of h1 and h2 is shown in Figure 7.

According to Figure 7, it is not difficult to find that the drilling temperature increases with the increase of spindle speed, but decreases with the increase of feed speed. When the feed speed is 50 mm/min, the drilling temperature of h1 and h2 increases by 27.08 °C and 20.82 °C, respectively, as the spindle speed increases from 1000 to 5000 rev/min. At this point, the temperature of h1 and h2 also rises to the highest point. This phenomenon is related to the friction between the tool and the workpiece. In addition, the increase in spindle speed causes more friction between the tool and the workpiece, causing more friction energy to be converted into heat. Moreover, the low thermal conductivity of PTFE makes it difficult to dissipate heat during drilling, which results in high temperatures. When the spindle speed is 1000 rev/min, the drilling temperature of h1 and h2 decreases by 9.31 °C and 16.94 °C, respectively, as the feed rate increases from 50 to 250 mm/min. At this point, the temperature of h1 and h2 also decreases to the minimum. This behavior can be attributed to the increase in feed rate which shortens the drilling time, and thus leads to a reduction in time of friction. Meanwhile, this prevents higher temperatures from being reached between the tool, the workpiece, and the chip.

**Figure 8:**
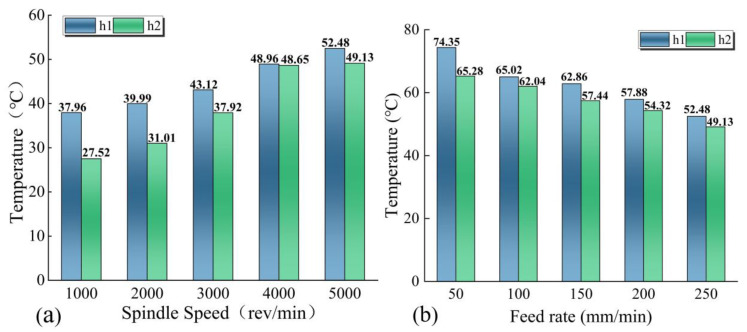
Comparative graphs of the temperatures of h1 and h2. (**a**) Spindle speed of 5000 rev/min; (**b**) Feed rate of 50 mm/min.

As can be seen from Figure 7, there is a significant temperature difference between h1 and h2. In order to compare the drilling temperature between h1 and h2 more visually, two sets of representative curves were plotted. Figure 8a shows the temperature comparison of two holes with different feed rates at a spindle speed of 5000 rev/min. The results obtained show that the temperature of h1 is always higher than h2 and the temperature of h2 decreases up to 12.2% compared to h1 as the feed rate increases. Figure 8b shows the temperature comparison of two holes with different spindle speeds at a feed rate of 50 mm/min. From the figure, it can be seen that the cutting temperature of h1 increases up to 45.32% compared to h2 as the spindle speed increases.

**Figure 9:**
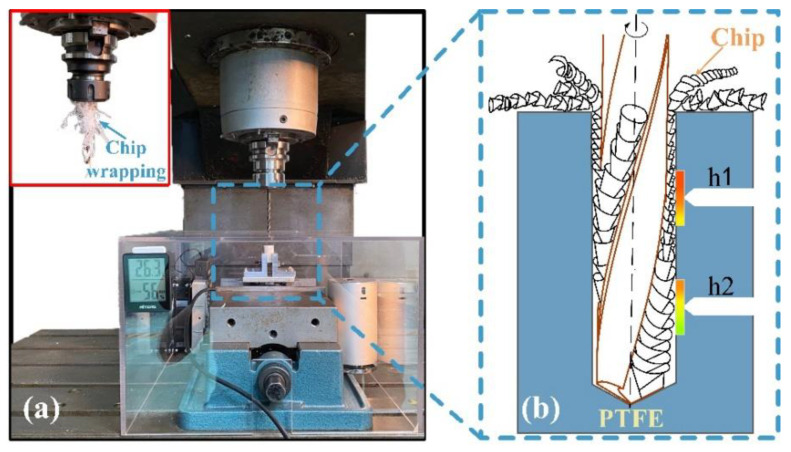
Chip accumulation in drilling of PTFE: (**a**) Chip accumulation phenomenon; (**b**) Schematic diagram of chip accumulation.

The temperature difference between the two holes is related to chip accumulation during drilling. Figure 9 illustrates the phenomenon of chip accumulation in the drilling. Figure 9a shows that the chips are mostly wrapped around the drill bit in ribbons at the end of drilling. The diameter of the “chip pile” formed by winding near the shank is significantly larger than the part far from the shank. Figure 9b shows a schematic diagram of the chip accumulation phenomenon. In the drilling of PTFE, continuous long chips will be produced due to the excellent toughness of PTFE. These chips cannot be discharged in time during the machining process. With the increase of the processing depth, chips are produced continuously, and the chips that are not discharged will be pushed and squeezed by these chips. This causes the undischarged chips to move up the spiral groove and gradually wrap around the tool, causing chips to accumulate in the spiral groove. In addition, due to the large thermal expansion coefficient and elastic recovery of PTFE, the phenomenon of hole shrinkage could occur during the drilling. This can also lead to chip clogging in the upper part of the workpiece, resulting in chip accumulation.

The position of h1 is closer to the tool shank than h2, so the accumulation of chips at h1 is more serious than that at h2. The accumulation of chips makes it difficult for the cutting heat to be conducted out, resulting in an increase in the cutting temperature at h1. In addition, chip accumulation also increases the friction area between chip and hole wall. This results in increased frictional heat between the chip and the hole wall, which increases the drilling temperature. Therefore, the drilling temperature of h1 is always higher than that of h2 regardless of the drilling parameters.

### 3.3. Effect of Drilling Parameters on Surface Roughness

**Figure 10:**
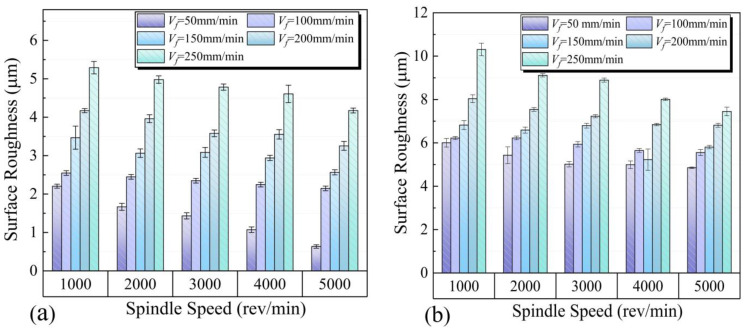
Surface roughness of hole inner wall under different cutting parameters: (**a**) Surface roughness of hole entrance; (**b**) Surface roughness of hole exit.

To illustrate the effect of drilling parameters, the variation of surface roughness near the hole entrance and hole exit with drilling parameters is shown in Figure 10. It can be seen in Figure 10 that the surface roughness of the hole entrance and hole exit decreases with the increase of spindle speed and increases with increasing feed speed. When the spindle speed is 5000 rev/min and the feed speed is 50 mm/min, the minimum surface roughness of the hole entrance and hole exit is 0.64 µm and 4.85 µm, respectively. The maximum values of surface roughness of hole entrance and hole exit are, respectively, 5.29 µm and 10.3 µm, achieved in the case of spindle speed of 1000 rev/min and feed rate of 250 mm/min.

The trend of surface roughness is consistent with the thrust under the influence of different drilling parameters. This is because thrust causes plastic deformation of the material, and increasing plastic deformation results in higher surface roughness [26,27].

It can be seen from Figure 10 that the surface roughness at the hole exit is always higher than that of the hole entrance. There are two reasons for the above phenomenon. First, the thickness of the uncut material changes with the feed of the tool. When the cutting edge of the tool first cuts into the PTFE, the uncut material is thick enough to resist the bending and vibration of the workpiece. At this stage, the machining vibration is very small or even negligible, so the machined surface quality is good at this time. As the tool cuts further into the workpiece, the uncut material becomes thinner. At this stage, the cutting vibration became quite strong and becomes the most important factor affecting the stability of the tool–workpiece system. The strong cutting vibration makes the tool-workpiece coupling stability extremely poor, which in turn deteriorates the machined surface and leads to increased roughness.

RMSE (root mean square error) is an important dimensional eigenvalue to evaluate the characteristics of signals in the time domain. The RMSE values of thrust could be used to evaluate the cutting stability in the machining process [28]. The stage at which the main cutting edge of the bit participates in cutting to a drilling depth of 10 mm is referred to as the initial drilling phase. The stage from the drilling depth of 20 mm to the drilling out of the bit is called the late drilling phase. Table 9 shows the RMSE values of thrust during the initial drilling phase and late drilling phase under different parameters.

As can be seen from the table, the RMSE at the late drilling phase was always greater than that at the initial drilling phase. This indicated that the cutting stability at the late drilling phase was not good as at the initial drilling phase. Therefore, the degree of machining vibration when drilling near the hole exit is much higher than that at the hole entrance. This results in a larger surface roughness at the hole exit than at the hole entrance.

**Figure 11:**
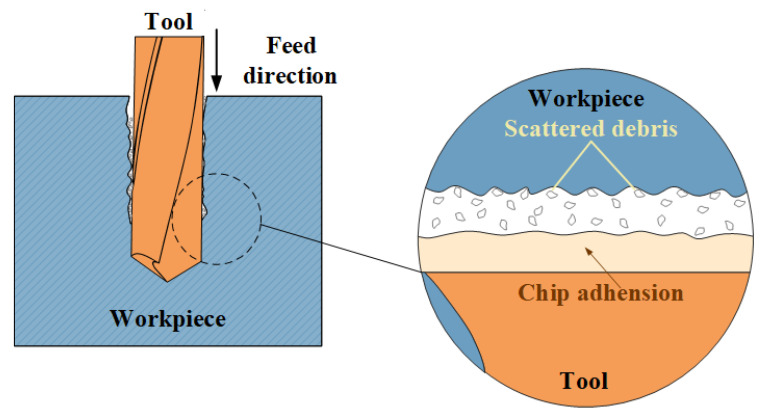
Mechanism diagram of the effect of chip adhesion on surface roughness.

Secondly, the chip that adhered to the bit during drilling has a very important effect on the surface roughness of the inner wall of the hole. In addition to generating continuous chips, a lot of chippings are generated during the drilling of PTFE. Due to chip clogging during drilling, a portion of the chippings are recaptured and ground. The chips adhere to the cutting edge of the tool due to the high temperature generated during the grinding process and the compression of the hole wall. Figure 11 illustrates the mechanism diagram of the influence of chip adhesion on surface roughness during drilling. Chippings adhering to the cutting edge of the drill act like a film, further hindering the cutting edges of the drill from machining the inside of the hole, resulting in poorer surface quality. In addition, there will be a small portion of chippings scattered between the drill bit and the inner wall of the hole. These chippings constantly rub against the machined surface, which also deteriorates the surface quality. It is considered to be that the large difference in surface roughness between the hole entrance and hole exit is related to the degree of chip adhesion on the tool.

Relevant drilling experiments were conducted to further verify our speculation. Two identical drill bits were used for drilling at different depths and the degree of chip adhesion of the two bits was observed. Two drilling depths of 10 mm and 30 mm were set in the experiment. The drilling depths of 10 mm and 30 mm were chosen to simulate the situation of the drill bit at the hole entrance and hole exit, respectively. After the experiment was completed, images were taken of two sets of drills at different drilling depths.

**Figure 12:**
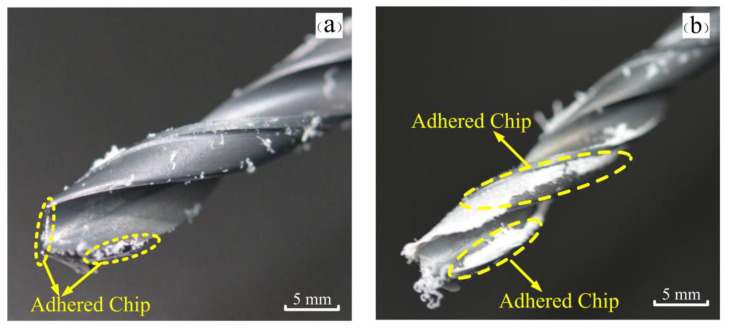
Pictures of chip adhesion at different drilling depths: (**a**) Drilling depth of 10 mm; (**b**) Drilling depth of 30 mm.

Figure 12 shows the degree of chip adhesion at different drilling depths. The degree of chip adhesion at a drilling depth of 10 mm is shown in Figure 12a. It can be seen from the figure that the main cutting edge and the secondary cutting edge of the drill have chip adhesion, but the degree of chip adhesion is not very serious, and the chips adhere to each cutting edge intermittently. The degree of chip adhesion at a drilling depth of 30 mm is shown in Figure 12b. We can observe that the chip adhesion phenomenon on each cutting edge of the drill is more obvious, and the degree of adhesion is more serious at this depth. Some chips coat the drill bit like a film. The degree of chip adhesion at the 30 mm drilling depth is more serious than that at 10 mm, which leads to a decrease in the shear effect of the main cutting edge of the drill, thereby reducing the surface quality. This experiment further confirms that the large difference in surface roughness at the hole entrance and hole exit is caused by chip adhesion.

## 4. Conclusions

In this study, the drilling experiments of PTFE were conducted by using an HSS twist drill. The influence of drilling parameters on the drilling process was evaluated in terms of thrust, torque, temperature, and surface roughness. The following conclusions were drawn from the present study.

(1) The decrease of the feed speed and the increase of the spindle speed will reduce the thrust and torque. The lowest value of thrust and torque were 22.64 N and 0.12 Nm when the spindle speed and feed rate were 5000 rev/min and 50 mm/min.
(2) The results of the ANOVA showed that the spindle speed contributed the most to the thrust at 46.8% and the feed rate contributed the most to the torque at 65%. The developed model can effectively predict the output response. The maximum error between the predicted values and actual values of thrust and torque is 10.7% and 13.7%, respectively.
(3) There are significant differences in temperature at different drilling depths during drilling. The difference is related to chip accumulation during the drilling of PTFE. Since PTFE is a thermoplastic polymer with high toughness, the chips generated during drilling are not easy to break, resulting in chip accumulation. Chip accumulation causes cutting heat to build up in the workpiece. The low thermal conductivity of PTFE makes it difficult for the cutting heat to conduct away, resulting in temperature rise. The temperature difference is caused by the different degrees of chip accumulation at different drilling depths.
(4) In the processing of PTFE, the surface roughness of the inner wall of the hole is not consistent, especially since there is a big difference between the entrance of the hole and the exit of the hole. This phenomenon is associated with chip adhesion during drilling. The adhered chips prevent the cutting edge from fully machining the inner wall of the hole, resulting in poor surface quality. with the increases in drilling depth, the degree of chip adhesion on the bit becomes more severe. Moreover, drilling PTFE with higher spindle speed and lower feed rate will provide better surface quality.

## Figures and Tables

**Table 1:** Physical and mechanical properties of the workpiece.

Properties	Values
Density	2.2 g/cm^3^
Melting temperature	327 °C
Glass transition temperature	130 °C
Thermal conductivity	0.25 W/(m·K)
Coefficient of linear expansion	10 × 10^−5^/°C
Dynamic friction factor	0.10
Bending elastic ratio	550 Mpa
Compressive strength	11.8 Mpa

**Table 2:** The geometrical parameters of twist drill.

Geometrical Parameters	Value (°)
Helix angle (β)	28
Relief angle (γ)	16
chisel edge angle (Ψ)	50
Rake angle (α)	30

**Table 3:** Cutting parameters of the validation experiments.

f (mm/min)	*n* (rev/min)				
	1000	2000	3000	4000	5000
50	H01	H02	H03	H04	H05
100	H06	H07	H08	H09	H10
150	H11	H12	H13	H14	H15
200	H16	H17	H18	H19	H20
250	H21	H22	H23	H24	H25

**Table 4:** Chip morphology under different machining parameters.

NO	Drilling Parameters		Chip Morphology
1		*f* = 50 mm/min	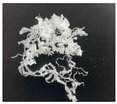
2	*n* = 1000 rev/min	*f* = 150 mm/min	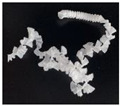
3		*f* = 250 mm/min	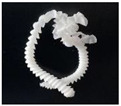
4		*f* = 50 mm/min	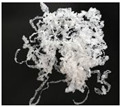
5	*n* = 3000 rev/min	*f* = 150 mm/min	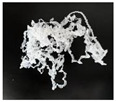
6		*f* = 250 mm/min	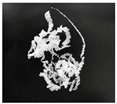
7		*f* = 50 mm/min	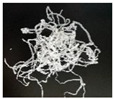
8	*n* = 5000 rev/min	*f* = 150 mm/min	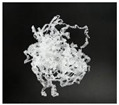
9		*f* = 250 mm/min	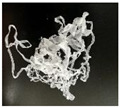

**Table 5:** ANOVA results for thrust.

Source	SS	DF	MS	F-Value	*p*-Value	Cont (%)
Model	6709.86	4	1677.46	279.49	<0.0001	
A, Spindle speed	3197.62	1	3197.62	532.78	<0.0001	46.8
B, Feed rate	2716.66	1	2716.66	452.64	<0.0001	39.8
AB	378.84	1	378.84	63.12	<0.0001	5.5
A^2^	416.75	1	416.75	69.44	<0.0001	6.1
R-square = 0.9824; Adjusted R-squared = 0.9789						

**Table 6:** ANOVA results for torque.

Source	SS	DF	MS	F-Value	*p*-Value	Cont (%)
Model	1319.09	1	439.70	176.67	<0.0001	
A, Spindle speed	365.58	1	365.58	146.89	<0.0001	26.7
B, Feed rate	891.43	1	891.43	358.17	<0.0001	65
B^2^	62.08	1	62.08	24.94	<0.0001	4.5
R-square = 0.9619;Adjusted R-squared = 0.9564						

**Table 7:** Validation experiments for thrust.

No	Spindle Speed	Feed Rate	Thrust (N)		Error
	(rev/min)	(mm/min)	Experimental	Predicted Value	(%)
1	1000	50	38.59	41.37	7.20
2	1000	250	85.90	86.43	0.62
3	3000	50	26.20	23.40	10.7
4	3000	250	53.43	52.89	1.01
5	5000	50	22.64	24.95	10.2
6	5000	250	39.04	38.87	0.44

**Table 8:** Validation experiments for torque.

No	Spindle Speed	Feed Rate	Torque (N)		Error
	(rev/min)	(mm/min)	Experimental	Predicted Value	(%)
1	1000	50	26.05	22.47079	13.7
2	1000	250	39.35	39.35963	0.01
3	3000	50	15.79	17.06279	8.1
4	3000	250	32.9	33.95163	3.2
5	5000	50	12.2	11.65479	4.5
6	5000	250	29.04	28.54363	1.7

**Table 9:** The RMSE values of thrust during different stages under different parameters.

NO	Spindle Speed (rev/min)	Feed Rate (mm/rev)	RMSE of Thrust	
			Initial Drilling Phase	Late Drilling Phase
1	1000	50	2.57625	2.87912
2		150	3.97652	4.11235
3		250	6.57521	6.75261
4		50	2.08725	2.76325
5	3000	150	3.57622	3.87215
6		250	6.01258	6.91205
7		50	1.87925	2.45630
8	5000	150	3.11250	3.01752
9		250	5.15752	6.12486

## Data Availability

Not applicable.
